# A Theory of Instrument-Specific Absolute Pitch

**DOI:** 10.3389/fpsyg.2020.560877

**Published:** 2020-10-22

**Authors:** Lindsey Reymore, Niels Chr. Hansen

**Affiliations:** ^1^School of Music, The Ohio State University, Columbus, OH, United States; ^2^Schulich School of Music, McGill University, Montréal, QC, Canada; ^3^Aarhus Institute of Advanced Studies, Aarhus University, Aarhus, Denmark; ^4^Center for Music in the Brain, Aarhus University, Royal Academy of Music Aarhus/Aalborg, Aarhus, Denmark

**Keywords:** absolute pitch, timbre, motor imagery, oboe, music, expertise

## Abstract

While absolute pitch (AP)—the ability to name musical pitches globally and without reference—is rare in expert musicians, anecdotal evidence suggests that some musicians may better identify pitches played on their primary instrument than pitches played on other instruments. We call this phenomenon “instrument-specific absolute pitch” (ISAP). In this paper we present a theory of ISAP. Specifically, we offer the hypothesis that some expert musicians without global AP may be able to more accurately identify pitches played on their primary instrument(s), and we propose timbral cues and articulatory motor imagery as two underlying mechanisms. Depending on whether informative timbral cues arise from performer- or instrument-specific idiosyncrasies or from timbre-facilitated tonotopic representations, we predict that performance may be enhanced for notes played by oneself, notes played on one’s own personal instrument, and/or notes played on any exemplar of one’s own instrument type. Sounds of one’s primary instrument may moreover activate kinesthetic memory and motor imagery, aiding pitch identification. In order to demonstrate how our theory can be tested, we report the methodology and analysis of two exemplary experiments conducted on two case-study participants who are professional oboists. The aim of the first experiment was to determine whether the oboists demonstrated ISAP ability, while the purpose of the second experiment was to provide a preliminary investigation of the underlying mechanisms. The results of the first experiment revealed that only one of the two oboists showed an advantage for identifying oboe tones over piano tones. For this oboist demonstrating ISAP, the second experiment demonstrated that pitch-naming accuracy decreased and variance around the correct pitch value increased as an effect of transposition and motor interference, but not of instrument or performer. These preliminary data suggest that some musicians possess ISAP while others do not. Timbral cues and motor imagery may both play roles in the acquisition of this ability. Based on our case study findings, we provide methodological considerations and recommendations for future empirical testing of our theory of ISAP.

## Introduction

Absolute pitch (AP), or the ability to identify or categorize musical pitches without external reference (Loui, 2016), is generally considered to be a rare skill. Although the etiology of absolute pitch remains under debate, current evidence suggests that AP emerges from interactions between innate and learned elements, leaving a role for both genetic predisposition and an early critical period for developing the ability (Loui, 2016). Estimates suggest that 0.01–1% of the population have absolute pitch (Ward, 1999; Lenhoff et al., 2001; Levitin and Rogers, 2005), and most expert musicians do not have what we refer to in this article as “global absolute pitch,” or the ability to name pitches across timbres without external reference. Yet, anecdotal evidence suggests that at least some musicians are better able to name notes that are played on their instrument of expertise than notes played on other instruments. We refer to this ability as “instrument-specific absolute pitch” (ISAP). In this study, we hypothesize that some expert musicians without global AP may demonstrate instrument-specific absolute pitch for their primary instrument(s) at levels that are above chance but do not necessarily reach perfection. By “primary” instrument, we mean the musical instrument that a musician has the most familiarity with and plays most regularly.

Better performance on pitch identification tasks for certain timbres over others has been established for global AP possessors in that accuracy of pitch identification decreases from natural complex tones to pure sine tones (e.g., Miyazaki, 1989; Athos et al., 2007; Hsieh and Saberi, 2009; Vanzella and Schellenberg, 2010; Gruhn et al., 2018). Furthermore, a sub-type of absolute pitch, “absolute piano,” has been posited to describe those individuals who can only identify pitches played in the piano timbre (Lockhead and Byrd, 1981; Levitin and Rogers, 2005). For global AP possessors who were violinists, Brammer (1951) observed increased accuracy for violin tones over clarinet tones when participants were asked to adjust tones to a frequency of 440 Hz corresponding to the note A4. Sergeant (1969) reports advantages for both those with and without global AP for notes from the first instrument they learned, with the highest accuracy found for instruments learned in early childhood. However, no quantitative data were reported in relation to this claim, and the methodology is not described, making it difficult to assess the prevalence or strength of this effect.

While most studies of absolute pitch have concentrated on individuals with near-perfect accuracy in pitch labeling, other research has suggested that many musicians have pitch-labeling abilities somewhere in the middle of a continuum of pitch identification accuracies (Levitin and Rogers, 2005; Wilson et al., 2009; Wengenroth et al., 2014; Leite et al., 2016). This intermediate ability has been called “quasi-absolute pitch,” and while exact definitions vary, generally refers to accuracy above chance but below around 85%. Wilson et al. (2009) observed behavioral as well as functional and structural brain differences among absolute pitch, quasi-absolute pitch, and relative pitch possessors. In analyzing qualitative descriptions of pitch-naming strategies from the participants with quasi-absolute pitch, several subgroups of responses were observed that implicated timbre, including self-report that identification was “best for familiar instrumental timbres in the middle range” (p. 728). This suggests a possible connection with instrument-specific absolute pitch as presented here.

In musicians without global AP, only a few studies have directly addressed the possibility that there is an advantage for one’s primary instrument. Wong and Wong (2014), for example, tested violinists and pianists, comparing pitch identification performance with sine tones and with tones played on their respective instruments and did indeed find an overall trend for increased accuracy in identifying pitches played on participants’ own instrument type over sine tones. However, since musicians were not tested on tones played on their non-primary instrument, this could be due to the use of complex tones rather than instrument-specific timbres. Marvin and Brinkman (2000) contrasted primary and non-primary instruments more directly with synthesized violin and piano tones. While they observed that pianists had an advantage for piano over violin tones, performance for violinists was not significantly different between the two instruments. Importantly, the absence of impaired performance for piano tones in violinists could be explained by their presumed familiarity with piano timbre, given that string players of Western classical music are typically expected to learn basic piano skills and are often trained and tested using piano sounds in the theory and aural skills curriculum. In a recent study, Li (2020) found no string advantage for string majors. However, although Li’s participants did not self-identify as either AP possessors or AP non-possessors, they were mostly musicians with very high degrees of global AP ability who started musical training before 9 years of age. Therefore, only a few of them would be expected to capitalize on mechanisms used by possessors of instrument-specific absolute pitch as proposed in this paper. Additionally, in a sample of 12 non-AP musicians, all of whom played the piano, but only some of whom played various other instruments, Schlemmer et al. (2005) only found statistically significant above-chance performance for white-key notes played in a familiar timbre. Yet, despite these efforts, no studies have investigated primary instrument advantages in wind, brass, or percussion players. Wong and Wong (2014) propose that the multimodal nature of musical experience contributes to pitch-naming abilities in musicians with intermediate levels of AP; however, no research has offered a theory of the specific mechanisms behind ISAP.

In this paper, we propose a theory of ISAP, suggesting that timbral cues and motor imagery contribute to increased pitch identification accuracy for musical instruments for which one has substantial motor and/or timbral experience. In order to demonstrate how our theory can be tested, we further report the methodology and exemplary analysis of two preliminary case studies testing for the existence and underlying mechanisms of ISAP.

## The Theory

Why might musicians be able to identify pitches played on their main instrument better than pitches played on other instruments? In the case of global AP, AP possessors are usually able to label pitch chroma independent of timbre, though, as we have seen, complex tones such as those played on the piano facilitate higher accuracy in comparison to sine tones. While a variety of other types of mechanisms may be at play in global AP, we propose that the mechanisms for instrument-specific absolute pitch (ISAP) are likely to be a product of the extraordinary timbral and kinesthetic familiarity that expert musicians develop with their own instrument.

### Timbral Cues

Musicians tend to be extremely familiar with the timbral peculiarities of their primary instrument type but not necessarily with other instruments (Margulis et al., 2009), with the caveat that many formally trained Western musicians have at least some experiential familiarity with the piano as well. Neuroscientific studies support the idea that extreme familiarity with a primary instrument has a marked impact on auditory processing. Pantev et al. (2001) found that for musicians, auditory cortical representations of trumpet and violin tones were enhanced preferentially for musicians’ primary instrument timbres. Shahin et al. (2008) demonstrated that violinists and pianists show enhanced gamma band activity when listening to timbres closest to their own instrument. Using fMRI, Margulis et al. (2009) identified brain areas in violinists and flutists that are selectively activated only when listening to the timbre of their own instrument. Thus, we propose that it is possible that increased or better coordinated cortical processing of primary instrument sounds may facilitate absolute pitch identification in a timbre-selective manner.

Learned differences in timbre and intonation among the notes afforded by an instrument may also contribute to the apparent learned ability to identify notes on a highly familiar instrument. These factors (timbre and intonation) can co-vary with pitch on at least three dimensions.

First, timbre and intonation tendencies^1^ sometimes change from low to high pitch, corresponding to physical changes in the length, for example, of a string or an air column (Sergeant, 1969; Siedenburg and McAdams, 2017). Previous research suggests that pitch differences over an octave make it harder to perceive sounds as coming from the same instrument (Handel and Erickson, 2001), though musicians seem to be more successful with this task than non-musicians, suggesting that musical training plays a role (Steele and Williams, 2006). Complications arising from the interaction of pitch and timbre have also been observed in speeded classification (Krumhansl and Iverson, 1992), and shifts in timbre appear to impair recognition memory for melodies (Schellenberg and Habashi, 2015). An experiment reported by Allen and Oxenham (2014) found interference of pitch and timbre, with results suggesting that in particular, neural processing of pitch height and timbral brightness are tightly related. Finally, evidence in support of a general link between perceptions of pitch and timbral brightness has been found in multiple studies (e.g., Marozeau and de Cheveigné, 2007; Cousineau et al., 2014; Allen et al., 2017). Intonation may also vary with pitch height. String instruments tend to play sharper in upper registers than in lower registers (Kantorski, 1986), and some singers show a similar tendency (Sundberg, 2011). While such changes may be predictable, the ways in which timbre and intonation change across an instrument’s range are likely different among different types of instruments. For example, deviance from equal temperament across the tessitura seemed more pronounced on the double bass than on other string instruments, and whereas intonation was generally sharper in the upper compared to the lower register for violinists, violists, and bassists, cellists appeared to show the opposite pattern with greater sharpness in the lower compared to the upper register (Kantorski, 1986).

Second, in addition to varying in pitch from low to high, timbral and intonation-related differences across an instrument’s pitch range may be further affected by an instrument’s register. The term “register” refers to a section of an instrument’s range, which often is referred to as having characteristic timbral qualities. Pitch itself is a continuous variable; register, as a concept often applied to an instrument’s range, is categorical. Different registers of a given instrument or voice are not determined by a fixed interval, but may be related to the construction of the instrument or the way in which the voice produces a pitch (Drabkin, 2001). Consequently, register as we discuss it here is defined relative to a single instrument type and is not constant across instruments—the lowest register of the flute, for example, overlaps with the highest register of the bassoon. Anecdotally, for some instruments more so than others, timbre varies characteristically among registers. For example, the clarinet’s range is typically described as comprising four registers: the dark, hollow “chalumeau” register from E3 to G4, the “throat” register from G#4 to B♭4, the brighter, sweeter “clarion” register from B4 to C6, and the shrill, “extreme” register above C6 (Page et al., 2001). Furthermore, intonation tendencies may interact with register independently from pitch height: for the clarinet, a tendency to go sharp at soft dynamics and flat at loud dynamics is more pronounced in the lower two than in the upper two of these registers (Raasakka, 2017), and notes tend to go increasingly flat at the extreme top of the highest register (Gangl and Hofmann, 2015).

Even for instruments without widely accepted vocabulary and definitions for specific registers, regions of the instruments often have distinct timbral characters. Colloquially, and for practical purposes, it is often common to refer generally to the low, middle, or high register of an instrument. For example, the Vienna Symphonic Library (n.d.) describes the French conservatory oboe’s registers as lower (B♭3–F4), middle (F#4–B♭5), and upper (B5–G6). Notes above G6 are possible but rare, and are considered the altissimo register (Redgate, 2018). The term “*altissimo*” generally refers to the extreme high register of any instrument. Notes in the *altissimo* register are usually not employed commonly in most music (though they are often relatively more common in modern and contemporary music) and often require high levels of skill to control. While the timbral characters of different registers of instruments are familiar to many musicians, and especially familiar for a player of a given instrument, no research has yet sought to systematically map timbre of instrument registers.

With natural instrument sounds, the relative contributions of pitch and register to an instrument’s timbre are difficult, if not impossible, to perceptually parse. However, there is some evidence that register may contribute uniquely to timbre, apart from variation in pitch: a machine learning model using Linear Discriminant Analysis (LDA) reported by Weihs et al. (2005) demonstrates highly successful classification of register by spectrum only, that is, when all pitch information has been eliminated.

The third dimension on which timbre and intonation can co-vary with pitch concerns pitch-specific timbral and intonation-related idiosyncrasies unique to certain instrument types that may be recognizable by expert musicians. For example, C5 on the oboe tends to be notoriously nasal, bright, and unsteady. The original (“forked”) fingerings for F4 and F5 lead to notably muffled-sounding timbres, to the extent that many professional oboists avoid these standard fingerings entirely, opting for fingerings producing timbres more consistent with the remainder of the instrument. Intonational idiosyncrasies also exist; for example, E5 on many oboes tends to be sharp relative to equal temperament, and in some contexts, oboists will add the low B key to help lower the pitch. The particular pattern of intonational tendencies for an instrument may vary slightly among brands, models, and musicians, but general tendencies for instrument types can be observed (as cataloged by Snow, 2006). For example, despite vast inter-individual differences in intonational tendencies observed in 35 trumpeters, A3 and A4 were consistently flat by 17 cents (i.e., 17 hundredths of an equal-tempered semitone) and sharp by 15 cents on average (Bertsch, 1998).

Such timbral and intonation-related idiosyncrasies are products of the physics and design features of the instruments. While the perceptibility of certain idiosyncrasies may vary slightly from brand to brand, many musicians will agree that higher-quality instruments tend to be more even in timbre than lower-quality instruments. Typically, as musicians gain experience, they become better able to maintain timbral evenness across the range of their instrument. Yet, pitch-specific idiosyncrasies may still be perceptible to musicians who are intimately familiar with that type of instrument, and thus they may potentially be useful in pitch identification. It may be that musicians encounter especially informative cues on idiosyncratic timbre and intonation for individual notes early on during their training. This makes timbral cues a likely candidate for an underlying mechanism of ISAP. Even though these timbral inequalities are deliberately minimized in expert-level performance, they are often still perceptible, particularly to expert listeners.

Note that different types of instruments likely diverge on how much timbre and intonation tendencies vary within each of these three dimensions. Timbral differences as a function of pitch height may be more or less noticeable depending on the type of instrument being played; for example, the difference in timbre between B♭3 and B♭4 might be greater on one instrument than on another. Additionally, certain instruments, like the clarinet, have more distinct categorical registers than others. Narrower categorical registers (i.e., spanning a smaller interval) may be advantageous in ISAP, as there is statistically a greater likelihood of accuracy once the register is correctly identified. Instrument types also vary on how many perceptible pitch-specific idiosyncrasies they possess. For example, Snow (2006) lists eight notes on the trombone with intonational tendencies, roughly 20% of the trombone’s range; however, 24 notes are listed as having tendencies on the oboe, roughly 70% of the oboe’s range. If ISAP relies in part on timbral cues, instruments with higher total variance across all three dimensions of timbre and intonation tendency variation may be more likely to support the development of ISAP. That is, if the oboe and trombone were roughly equal on the first two dimensions (i.e., overall pitch range and characteristic registers), we would anticipate that oboists would be more likely to have ISAP than trombonists, as the oboe has much higher variance on this third dimension (i.e., pitch-specific idiosyncrasies). In addition, if ISAP is a product of implicit learning of registral and/or idiosyncratic differences, we would expect that instrument-specific timbral and intonational variability would predict how accurately individual pitches are identified on average by players of that particular instrument type.

Idiosyncratic intonation tendencies will result in tunings with more unique interval content. It is well-known that tonality perception and the historical development of musical scales have been guided by the (possibly implicit) identification of unique intervals and interval combinations (Browne, 1981; Butler, 1983; Gauldin, 1983; Huron, 1994); while this phenomenon has primarily been studied in the context of identifying modes and scale degrees within equal-tempered tuning systems, it is not impossible that subtle, yet stereotypic, deviations from equal temperament may aid in pitch identification for musicians who are especially familiar with these instruments. This would particularly be the case for certain string and wind instruments where the musician has greater control over sub-semitone intonation than in, for example, the case of the piano.

While the piano has been the instrument most often studied with respect to pitch-naming ability, its modern design and equal temperament tuning make it likely one of the instruments that varies the least on note-specific timbre/intonation: an equal-tempered and properly tuned piano in principle has no other tendencies than those possibly resulting from overall differences in string length, thickness, and the number of strings associated with each single pitch. Hearing and remembering timbral cues may thus be relatively easier with an instrument such as the oboe, which displays a higher number of idiosyncratic tendencies and a smaller overall pitch range. After the piano, the violin has been studied second-most often; yet string instruments also would seem to offer less timbral variation than the oboe, as we speculate that tone production is more equal across the range of a string instrument than on an oboe, despite timbral differences across different strings.

As timbral and intonation-related idiosyncrasies arise from aspects of the material, design, and physical construction of the instrument, there is a possibility that some idiosyncrasies will be shared by members of the same instrument family. Yet, there are vast differences in tone production between, for example, flutes, single-, and double-reeds, and even members of the string family differ noticeably in tessitura, size, shape, string thickness, bow, and tone production. In any case, idiosyncrasies will differ considerably more between than within instrument types. This suggests that finding empirical evidence for ISAP may be facilitated by first focusing on musicians who play more timbrally idiosyncratic instruments like the oboe rather than more timbrally even instruments like the piano. In fact, if timbral and intonational idiosyncrasies provide relevant information for identifying pitch, ISAP might be learnable by non-musicians, and some musical instruments may better enable ISAP than others. This would also provide a way of assessing whether timbral cues can facilitate ISAP independently from motor cues.

In sum, our first hypothesis about the mechanisms of ISAP is that learned instrument type-specific timbral variations in register and across individual pitches or fingerings aid musicians in identifying pitches played on their own instrument type. Two corollary hypotheses arise from this proposition. First, if musicians are sensitive to trends in timbral variation on an instrument type in general, it is possible that they can use even more specific idiosyncratic cues from additional levels of familiarity with a particular token instrument to identify pitches. Most musicians, with the possible exception of keyboard players, play almost exclusively on a single instrument. As each instrument varies subtly in tone color and intonational or timbral tendencies, musicians may be particularly attuned to the idiosyncrasies of their personal instrument. Whipple (1903) reports a case study in which an AP possessor performed significantly better in pitch identification on her own piano than on the experimenter’s piano (92% vs. 70% accuracy). In this case, we would expect musicians without AP to have an advantage in identifying pitches played on their personal instrument over pitches played on a different instrument of the same type.

Additionally, musicians spend the majority of their daily practice time listening to their own individual tone and playing style. While subtle differences among the tones of a group of musicians playing the same instrument may not be immediately apparent to outside listeners, there are perceptible variations in sounds among players (Chudy, 2016). Through exposure, musicians may learn to recognize another musician’s tone as you would be able to recognize the voice of someone you know well; for example, music teachers may be able to identify the sounds of their students even in blind auditions. Thus, the second corollary hypothesis proposes that additional levels of familiarity with one’s own, personal tone would aid musicians in instrument-specific absolute pitch identification. If this is the case, we would expect that musicians would better be able to identify pitches that they themselves recorded than pitches recorded by another person.

### Motor Imagery

Aside from perception and recognition of pitch-related timbral idiosyncrasies, we propose a second mechanism for ISAP: articulatory motor imagery. That is, pitch recognition may be linked to the learned connections between sounds and the kinesthetic actions required to create those sounds. While playing their instruments, musicians make constant connections between the note that is being sounded and the motor program required to execute that note on the instrument (D’Ausilio et al., 2006; Zatorre et al., 2007). For wind players, this kinesthetic connection is not only with the requisite fingering, but also with the shape and pressure of the embouchure. We theorize that upon hearing a note played on their primary instrument, the motor areas of the brain involved in producing that sound on the instrument are activated. This kinesthetic memory aids the musician in identifying pitches on their primary instrument.

While music is known to activate the motor system in the brain across individuals, differences in this activation have been observed between musicians and non-musicians. Burunat et al. (2015) observed increased functional symmetry during music listening in musicians compared to non-musicians in brain regions involved in somatosensory and motor control; differences in functional symmetry were also found between piano and string players. Furukawa et al. (2017) found that expert pianists demonstrated muscle-specific M1 excitability in response to listening to synthesized piano tones while non-musicians did not. Hou et al. (2017) observed that brain regions believed to house mirror neurons showed significantly more activation for pianists than non-musicians in response to viewing motion capture piano performances. More generally, Halpern et al. (2004) observed subthreshold activity in supplementary motor area (SMA) during timbre imagery tasks. Wallmark et al. (2018) demonstrated a motor component to timbre perception; they interpret their results as indicating a possible propensity to link timbral qualities with associated actions.

Especially pertinent to the current study, instrument-specific structural and functional changes have been observed. When comparing beatboxers, guitarists, and non-musicians, Krishnan et al. (2018) found that musicians showed enhanced sensorimotor activity only when listening to their primary instrument (beatboxing or guitar) but not when listening to the other instrument. Proverbio and Orlandi (2016) found differences in brain responses in musicians when listening to their own instrument timbre and listening to the timbre of an instrument they do not play. The authors show that effects of musical training can be instrument-specific, finding such effects in both specific visuomotor and audiomotor circuits.

Pitch-labeling in global AP possessors may be related to motor imagery for at least some people: in a study by Gruhn et al. (2018), a subset of participants described strong associations between absolute pitches and motor and body sensations, like instrumental fingerings or laryngeal position. Whipple (1903) describes the self-reported experience of an AP possessor identifying pitches as having a motor component: when listening to music, “finger movements are vaguely felt” (p. 291). Whipple concludes that the association of finger movements is a critical feature of the subject’s AP memory. However, global AP possessors who might call on kinesthetic memory are still able to generalize pitch across timbres, while the population we are interested in is less able to do this. Note that kinesthetic memory, in this case, can presumably only be evoked by the timbre of the relevant instrument and not by the pitch chroma itself. Specifically, if musicians with ISAP were only using chroma to label pitches, we would expect this ability to generalize across timbres.

If articulatory motor imagery contributes to ISAP, we would expect to see differences in the details of this mechanism in different types of instrumentalists. For example, most pitches played on the oboe map to only one fingering each, and the embouchure is also involved in maintaining intonation and tone. In the case of the trumpet, a single fingering may produce several different pitches, depending on the speed and direction of the air and the embouchure. For string instruments, no embouchure is involved, and a single pitch may be produced with multiple fingerings using different strings. Finally, pianists have no one-to-one mapping of pitch to fingering, as any pitch may be played by any finger depending on the musical context that it occurs in, and embouchure is not at all involved. In the case of piano, absolute pitch may thus be more successfully mapped to position in peripersonal space (cf. Kóbor et al., 2006) than to the execution of specific motor programs. These differences in how specifically pitch maps to kinesthetic patterns may affect how informative articulatory motor imagery is for identifying absolute pitch for different instruments.

**FIGURE 1:**
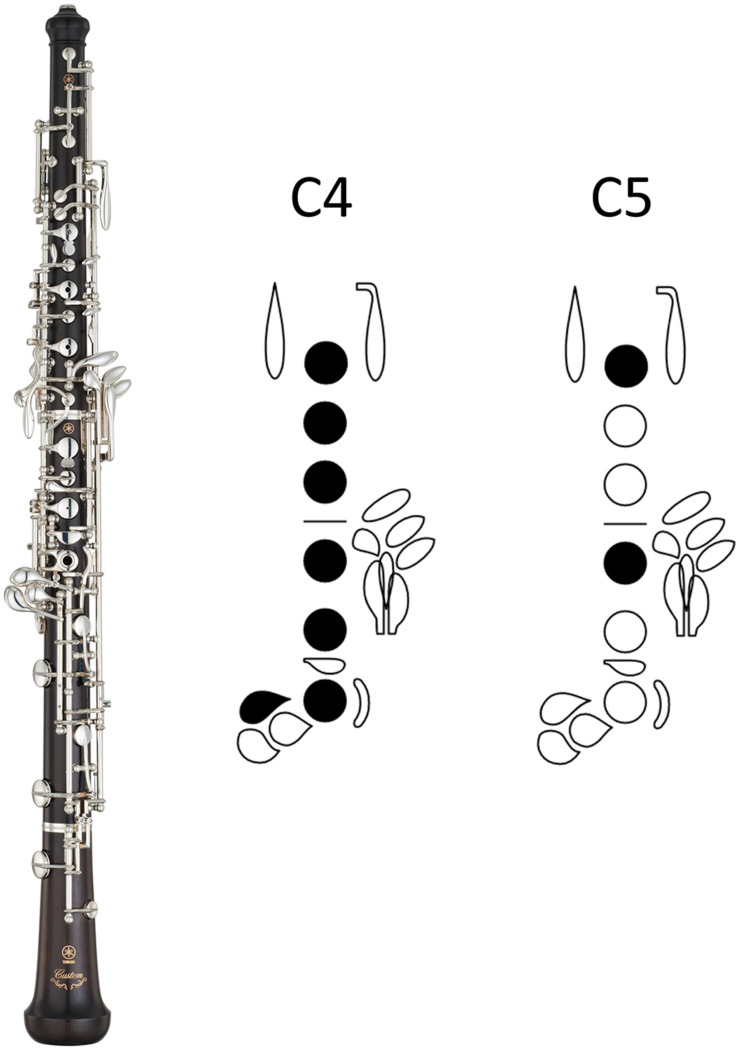
The oboe (left) and fingerings for C4 (middle) and C5 (right). Oboe photograph by Yamaha Music Europe. Graphic created via Fingering Diagram Builder (https://fingering.bretpimentel.com/).

While all Western instruments involve the use of hands (and often fingers) in motor imagery, wind instruments require refined control over combinations of fingerings, embouchure, and air speed to create the desired sound. For example, playing a C4 and a C5 on a piano can be accomplished by the same physical action, i.e., pressing the key with a finger. However, on the oboe, C4 and C5 require that a different set of fingers press the keys in combination (see Figure 1). Furthermore, on an American-style reed, a successful C4 requires a slightly more open jaw, relatively less lip pressure on the reed, and especially firm corners of the mouth, like a purse of the lips. For C5, slightly more reed is taken into the mouth by subtly rolling in the lips, and the jaw is not dropped as much as for the octave below. Firm corners of the mouth help to round and darken the tone, but the note will speak more easily than the low octave. Thus, we might again expect that the timbre of the oboe may provide more kinesthetic cues for expert oboists than piano timbre does for expert pianists, since more specialized motor areas are dedicated to producing specific pitches. Indeed, Choi et al. (2015) found that cortical thickness in experienced wind instrumentalists was significantly thicker in lip-related areas of the brain and thinner in tongue-related areas when compared to non-musician controls. They speculate that physical changes in the muscles involved with embouchure might drive changes in corresponding somatosensory brain regions over time. Such structural specificity may be related to instrument-specific absolute pitch. The fact that different instruments activate different motor and somatosensory areas entails that, in empirical studies of ISAP testing motor involvement, corresponding interventions will be needed in order to successfully interfere with this mechanism dependent on the muscles involved in sound production (and thereby sound perception) on a given instrument.

Thus, our theory of instrument-specific absolute pitch can be operationalized in terms of two primary mechanisms which may affect pitch identification accuracy. First, timbral cues may provide information on pitch to individuals with ISAP. We have considered three types of timbral cues that may be involved: (a) cues specific to a musician’s own, personal instrument, (b) cues specific to a musician’s own, idiosyncratic tone and playing style, and (c) pitch-specific idiosyncrasies that are common to instruments of a particular type (e.g., the oboe). Second, we propose that musicians implicitly use articulatory motor imagery when attempting to identify pitches played on their own instrument type.

## Case Studies

Two case studies were conducted in order to provide exemplary methodological guidelines on how the phenomenon of instrument-specific absolute pitch (ISAP) may be investigated in future research. Each case study comprised two tests (Experiment A and Experiment B). The purpose of Experiment A was to test for the presence of ISAP. The purpose of Experiment B was to test four operationalizations of the two proposed underlying mechanisms (timbral idiosyncrasies and motor imagery).

As no prior research has narrowly addressed ISAP or its underlying mechanisms, we needed to conduct these pilot studies to allow power calculations for future empirical studies. Because testing our four operationalizations in a full, factorial design required 16 conditions, the paradigm of Experiment A took approximately 2 h to complete, while the paradigm of Experiment B took a total of approximately 7 h to complete, spread out over the course of several days. Such a lengthy paradigm is impractical in many typical experimental settings. Our intention in beginning our investigation with case studies was to discover information that would allow future research to streamline the paradigm before ISAP and its mechanisms can be tested in a larger population. We offer these case studies in the current paper in order to demonstrate methodology and analysis that can be used to test our theory of ISAP and to suggest, based on the results, which aspects of our theory should be prioritized in future empirical testing.

### Participants

Both experiments were run on two professional female oboists trained in the United States. Oboist 1 (the first author) was 29 years old at the time of the experiment, had been playing the oboe for 19 years, and holds a bachelor’s and a master’s degree in oboe performance. Oboist 2 was 25 years old, had been playing the oboe for 13 years, and holds a bachelor’s degree in oboe performance. Both oboists had received similar formal training in piano (2 years of class piano during their undergraduate studies), although Oboist 1 had regularly used basic piano skills more often than Oboist 2 as an instructor for university aural skills and music theory. Oboist 1 began training in fixed-do solfège at age 18 and has since integrated it into her personal practice, though she teaches both fixed- and moveable-do; Oboist 2 was trained from the same age in moveable-do only and has not continued practicing it beyond the required 2 years. Both oboists confirmed that they did not have reason to believe they possessed global absolute pitch. Both oboists reported to be fully familiar with the Scientific Pitch Notation convention whereby letters designate note names and integers designate octaves (with middle-C labeled as C4).

### Stimuli

Stimuli were recorded by both oboists participating in the two experiments. Recordings were made using an AKG 414 microphone, set to cardioid pattern, through an API 3124 preamp, and the recording medium was Pro Tools, with an SSL Delta-Link interface. Recordings were normalized to −18 dBFS (decibel relative to full scale). For each condition, the oboist held individual notes for one half-note each at quarter note = 70 bpm (about 1.7 s, with durations spanning from 1.5 to 2.1 s). While recording, musicians watched a blinking metronome set to 70 bpm and were instructed to stop playing as the third blink occurred. Oboists were instructed to play musically and with their best tone at a comfortable and moderate dynamic level, using vibrato characteristic of their typical sound. While recording, the oboists referred to a tuner set at A4 = 440 Hz and were instructed to re-record tones that were not in tune.

Each oboist recorded the full set of available chromatic pitches from B♭3 to G6 in two conditions, once using their own oboe and once using the other oboist’s oboe. Oboists used their own reeds when recording with both instruments and were instructed not to change reeds between recordings. The rationale for this was that in the context of the present experiments, reeds were considered a property of the performer rather than of the instrument as each oboist has different personal preferences and customizes their own reeds.

All subsequent stimulus preparation and editing was carried out in Cubase 7.0.5 solely by the second author with no involvement of Oboists 1 and 2. Initially, the Split Function was used to segment recorded tracks into candidate single-tone clips starting at 1 s before tone onset with a total clip duration of 4 s. Excerpts with talking and/or not properly sounded tones were discarded. Pitch was confirmed using the Android app “Vocal Pitch Monitor.” There were at least two candidate clips available for each pitch level, but in some cases there were up to six clips because the oboist had repeated a given pitch to correct intonation or sound quality. A single preferred clip was selected for each pitch level by the second author (who is not an oboist) aiming for a balanced compromise among the following features: (a) full and harmonious tone; (b) singular, balanced, and non-noisy onset; (c) duration as close as possible to the intended; (d) minimal fluctuations in sound intensity level throughout the note; and (e) accurate intonation.

To produce the pitch-shifted stimuli used in Experiment B, each track was copied and manipulated using the Pitch Shift function in Cubase 7.0.5. Specifically, pitch was shifted up or down following Table 1 such that every consecutive set of eight pitches was transposed by +4, −1, +3, −2, +2, −3, +1, and −4 semitones leading to a new set with the same sounding pitches as the original one. This eightfold repeating pattern ensured that transpositions differed between consecutive octaves (which would not have been the case for four, six, or twelvefold patterns) while avoiding pitch-shifting of more than four semitones—which could have been easier to perceive due to continuous timbral differences across the range of the oboe. As 2 is the remainder after dividing 34 by 8, the pitches of the two highest tones G♭6 and G6 were simply exchanged through pitch-shifting. Pitch shifting used the Time Correction setting (to ensure that the duration of each clip stayed the same) as well as the Solo Musical setting, which represents a high-quality algorithm optimized for offline processing of monophonic musical material. Formant Preservation was not applied since pilot tests showed that this setting generated clearly audible artifacts giving rise to “whirling” sounds in the background noise before and after oboe tones.

**TABLE 1:** Template for generating the pitch-shifted oboe stimuli variants used in Experiment B.

Original pitch	Transposition (semitones)	Modified pitch
B♭3	+4	D4
B3	−1	B♭3
C4	+3	E♭4
D♭4	−2	B3
D4	+2	E4
E♭4	−3	C4
E4	+1	F4
F4	−4	D♭4
G♭4	+4	B♭4
G4	−1	G♭4
A♭4	+3	B4
A4	−2	G4
B♭4	+2	C5
B4	−3	A♭4
C5	+1	D♭5
D♭5	−4	A4
D5	+4	G♭5
E♭5	−1	D5
E5	+3	G5
F5	−2	E♭5
G♭5	+2	A♭5
G5	−3	E5
A♭5	+1	A5
A5	−4	F5
B♭5	+4	D6
B5	−1	B♭5
C6	+3	E♭6
D♭6	−2	B5
D6	+2	E6
E♭6	−3	C6
E6	+1	F6
F6	−4	D♭6
G♭6	+1	G6
G6	−1	G♭6

In addition to the 272 oboe files resulting from crossing the two-level factors Instrument (Oboe 1 vs. Oboe 2), Performer (Oboist 1 vs. Oboist 2), and Transposition (pitch shift vs. no pitch shift), 34 piano files were created corresponding to each of the pitch levels from B♭3 to G6, using “Acoustic Grand Piano” MIDI samples from the HALion Sonic SE library with a constant MIDI velocity of 127. This velocity value was chosen because it produced sound pressure levels in the final WAV files that were comparable to the ones for the oboe files. The duration of the piano tones corresponded to that of the oboe tones. All final oboe and piano tracks were exported from Cubase as WAV files at 44.1 kHz with 32 bits (float).

### Experiment A

#### Method

**FIGURE 2:**
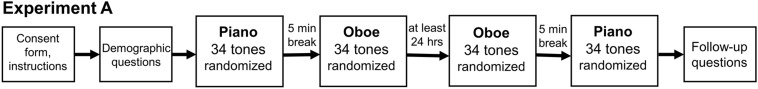
Experimental design for Experiment A, consisting of four blocks of 34 randomized tones each. Two blocks contained piano tones, while the other two contained oboe tones. Each oboist heard the tones that she herself had recorded on her own instrument.

The purpose of Experiment A was to determine if the two oboists were able to identify pitches played on the oboe more accurately than pitches played on the piano, spanning the full range of the oboe (B♭3–G6). To avoid carryover effects between oboe and piano tones where superior performance for one instrument could be used to guess the correct pitch of tones played on the other instrument via relative pitch kept in working memory, the stimuli were presented in separate piano blocks and oboe blocks presented in the reverse counterbalanced order piano-oboe-oboe-piano using the Qualtrics software (Qualtrics, Provo, UT, United States). The order of pitch levels was randomized anew for each block. The first two and last two blocks were completed on 2 separate days with a 5-min break between blocks completed on the same day. During Experiment A, each oboist only heard recordings of tones that they had recorded themselves on their own oboe. The 34 pitches within each block were presented in random order. For an illustration of the experimental design of Experiment A see Figure 2.

Instructions read: “In this study, you will listen to a series of single tones. For each tone you listen to, you will be asked to name the pitch that is played. You will be presented with a list of all of the available pitches from B♭3 to G6 and asked to select the one that is being played. You may play the recording of each note as many times as you wish. Please use headphones and take the survey in a quiet setting. Before starting, you should make sure to adjust the sound to a comfortable and audible level.” Oboists selected each pitch name from a list of all 34 possible pitches with both enharmonic equivalents listed when appropriate (e.g., F#4/G♭4). Participants were asked to provide free-form responses to four post-experiment questions designed to assess (1) whether they used any specific response strategies, (2) whether they experienced fatigue during the experiment, (3) whether they felt that some questions were harder than others, and (4) how confident they felt in their responses overall.

#### Results

**FIGURE 3:**
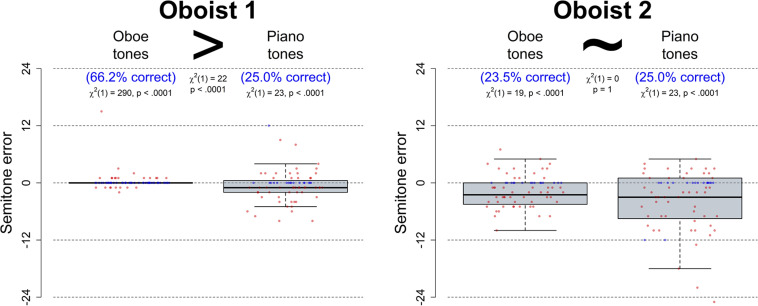
Boxplots with raw data of response deviation (in semitones) from the target pitch for correct (blue) and incorrect (red) pitch identification responses provided by the two oboists during Experiment A. Responses pertain to oboe tones (left) and piano tones (right), the former of which were recorded by the relevant oboist herself on her own instrument. Percentage of correct responses in each condition is indicated in blue writing, and the box plots depict the number of semitones that responses were off from the target. The presence and direction of significant differences between conditions—as assessed with Pearson’s chi-squared tests—are indicated with “>” signs whereas “∼” indicates that there was no significant difference between conditions. Whereas both oboists performed above chance for both oboe and piano tones, Oboist 1 furthermore identified pitch more accurately and made errors that were closer to the correct response for oboe compared to piano tones.

The results from Experiment A for each of the two oboists are depicted in the two sub-panels of Figure 3 with blue dots for correct trials and red dots for incorrect trials and the number of semitones off indicated on the y-axis. As evident from visual inspection, errors equal to or greater than an octave were very rare with no more than 8 out of 272 (2.9%) responses being 12 or more semitones off from the target. Similarly, octave confusions were rare in that no more than 3 out of 272 (1.1%) responses were off by exactly one or more octaves. This suggests that both oboists were largely correct in identifying the relevant octave register for both oboe and piano tones. Since, however, all octave confusions occurred for piano tones whereas no octave confusions occurred for oboe tones, we adopted the most conservative analysis strategy given our hypothesis pertaining to better absolute pitch identification for oboe over piano tones. Consequently, in the analysis reported below, responses that were off by exactly 12 or 24 semitones (i.e., octave confusions) were regarded as correct. Moreover, a chance level of 8.33% (i.e., 1/12) was adopted whereby octave equivalence was assumed. Importantly, due to the low number of octave-related errors, the statistics were nearly identical if octave equivalence was not assumed.

##### Accuracy.

One-sample proportions tests with continuity correction confirmed that performance was indeed significantly above chance for both oboists’ overall performance (Oboist 1: 45.6% correct, 95% CI: 37.1–54.3%, χ^2^(1) = 242.25, *p* < 0.0001; Oboist 2: 24.3% correct, 95% CI: 17.5–32.5%, χ^2^(1) = 43.13, *p* < 0.0001). Above-chance performance was also achieved when looking at oboe tones (Oboist 1: 66.2% correct, 95% CI: 53.6–76.9%, χ^2^(1) = 290.32, *p* < 0.0001; Oboist 2: 23.5% correct, 95% CI: 14.4–35.6%, χ^2^(1) = 18.62, *p* < 0.0001) and piano tones separately (Oboist 1: 25.0% correct, 95% CI: 15.6–37.2%, χ^2^(1) = 22.59, *p* < 0.0001; Oboist 2: 25.0% correct, 95% CI: 15.6–37.2%, χ^2^(1) = 22.59, *p* < 0.0001). Whereas Pearson’s chi-squared tests with Yates’ continuity correction showed that Oboist 1 was significantly more accurate for oboe than for piano tones (χ^2^(1) = 21.61, *p* < 0.0001), Oboist 2 did not demonstrate a significant difference in accuracy for oboe vs. piano tones (χ^2^(1) = 0, *p* = 1).

##### Semitone Error Values

Figure 3 suggests that Oboist 1’s responses for oboe tones were not only more correct than her own responses for piano tones or than Oboist 2’s responses for either instrument type, but they also seemed to exhibit smaller degrees of variance around the correct pitch value. Specifically, whereas nearly all of Oboist 1’s errors for oboe tones were within one semitone above or below the actual pitch (19 out of 23, 82.6%), this proportion was much smaller for Oboist 2 (11 out of 52, 21.2%). As data were not normally distributed, Mann-Whitney *U* tests were used to confirm that absolute semitone error values for incorrect responses were indeed significantly higher for piano tones than for oboe tones in the data from Oboist 1 (piano: Med = 2, IQR = 3; oboe: Med = 1, IQR = 0; *U* = 327.5, *p* = 0.0010) whereas this was not the case to a significant degree in the data from Oboist 2 (piano: Med = 3, IQR = 5.5; oboe: Med = 3, IQR = 3; *U* = 1076.5, *p* = 0.0969). Moreover, for oboe tones, absolute semitone error values were significantly lower for Oboist 1 than for Oboist 2 (*U* = 986.5, *p* < 0.0001). Finally, even though the proportion of correct responses for piano tones was identical for the two oboists (25.0%), the absolute semitone error values were still significantly lower for Oboist 1 than for Oboist 2 (*U* = 1809, *p* = 0.0005). This effect remained even if octave equivalence was assumed by taking modulo 12 of the absolute semitone error values (*U* = 1721, *p* = 0.0042).

Taken together, the findings from Experiment A suggest that whereas both oboists identified absolute pitches above chance level for oboe as well as for piano tones, only Oboist 1 showed evidence of ISAP. Furthermore, this skill was characterized not only by more correct responses but also by incorrect responses that were significantly closer to the correct pitch value. Curiously, while Oboist 1 provided pitch guesses for piano tones that were significantly closer to the correct pitch than those provided by Oboist 2, both oboists performed equally well in terms of overall accuracy for piano tones. Having established that ISAP is present in a musician who does not qualify as a global AP possessor, Experiment B was designed to assess the underlying mechanisms behind this ability.

### Experiment B

The purpose of Experiment B was to investigate whether instrument, player, pitch-shifting, and/or motor interference would affect the accuracy of ISAP. To test for the effect of pitch-specific timbral cues, accuracy was compared for original recordings and artificially pitch-shifted stimuli. If oboists use pitch-specific timbral cues to identify pitches, artificially shifting pitch should interfere with the ability to make accurate judgements.

To test for effects of familiarity with the timbral idiosyncrasies of their personal instrument, both oboists labeled pitches from recordings played on their own oboe as well as on the other person’s oboe. Likewise, to test for effects of performer-specific timbral cues, judgments were made for recordings of themselves playing as well as recordings of the other person playing.

To test for effects of motor imagery on ISAP, we developed an interference task that was expected to impair pitch-naming accuracy because it increased demands on motor-related brain areas involved in playing the instrument. In the case of the oboe, this would include the hands and fingers as well as lips and jaw, which are called upon for crucial embouchure adjustments while playing. Beaman et al. (2015) found that chewing gum has negative effects on spontaneous musical recollection (earworms), in support of the idea that chewing gum interferes with motor-related subvocalization or subvocalization-like processes that are linked to earworms. Consequently, a motor interference task was implemented in Experiment B.

#### Method

Using the stimuli described in the general Stimulus section above, Experiment B comprised a full factorial design crossing the following four two-level factors: instrument (own oboe vs. other oboe), performer (self vs. other), transposition (original vs. pitch-shifted), and motor interference (no interference vs. motor interference). The motor interference condition entailed two concurrent tasks which were performed by the oboists while listening to the stimuli and identifying pitches. Specifically, oboists were asked to chew gum as well as cross their arms near the wrists and continuously wiggle their fingers in a quick manner without a regular pattern.

**FIGURE 4:**
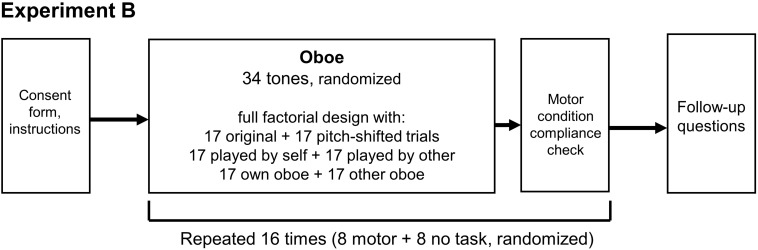
Experimental design for Experiment B, consisting of 16 blocks of 34 randomized tones each. Within each of the blocks, three of the conditions were balanced (self vs. other, own instrument vs. other, and pitch-shifted vs. original). Participants engaged in the motor tasks during 8 of the 16 blocks; the presentation order of motor vs. no motor blocks was randomized. For further detail on the distribution of trials within blocks see Supplementary Table 1.

Overall, Experiment B consisted of 16 blocks which were presented to each of the two oboists in random order using the Qualtrics software (Qualtrics, Provo, UT, United States). The order of pitch levels was randomized anew for each block. Eight blocks were completed with the motor interference task, and the other eight were completed with no interference task. These two block types were presented in random order for each participant. Across each set of 8 blocks, a total of 272 sound files represented three of the two-level factors (own vs. other instrument, self vs. other playing, and pitch-shifted vs. original). These 272 sound files were randomly distributed over 8 blocks so that each block consisted of 34 trials, one at each of the 34 chromatic pitch levels from B♭3 to G6, in random order (see Supplementary Table 1 for further details). Within each of the blocks, there was always an equal distribution of 17 original and 17 pitch-shifted trials, 17 trials performed by the participant herself and 17 trials performed by the other person, as well as 17 trials played on the participant’s own instrument and 17 trials played on the other person’s instrument. That is, eight blocks were devised to collectively comprise no more than a single, unique occurrence of all 272 sound files, and each of these eight blocks were completed once with the motor interference task and once without. For an illustration of the experimental design of Experiment B see Figure 4.

After each block, two validity check questions confirmed that participants always listened through headphones and complied with task instructions in terms of chewing gum and moving their fingers or refraining from doing so. Following the final block, the two participants were asked to provide free-form responses to the same post-experiment questions as in Experiment A.

#### Results

As for Experiment A, errors that were greater than or equal to 12 semitones off from the target were extremely rare, not occurring for Oboist 1 at all (0.0%) and occurring no more than 22 out of 544 times for Oboist 2 (4.0%). Octave confusions, in which the correct chroma was identified but assigned to the incorrect octave, also did not occur for Oboist 1 (0.0%) whereas 10 occurrences were present in the data from Oboist 2 (1.8%). Consequently, the approach from Experiment A was adopted whereby octave confusions were regarded as correct responses and the chance level was set to 1 out of 12 (8.33%).

##### Accuracy

**FIGURE 5:**
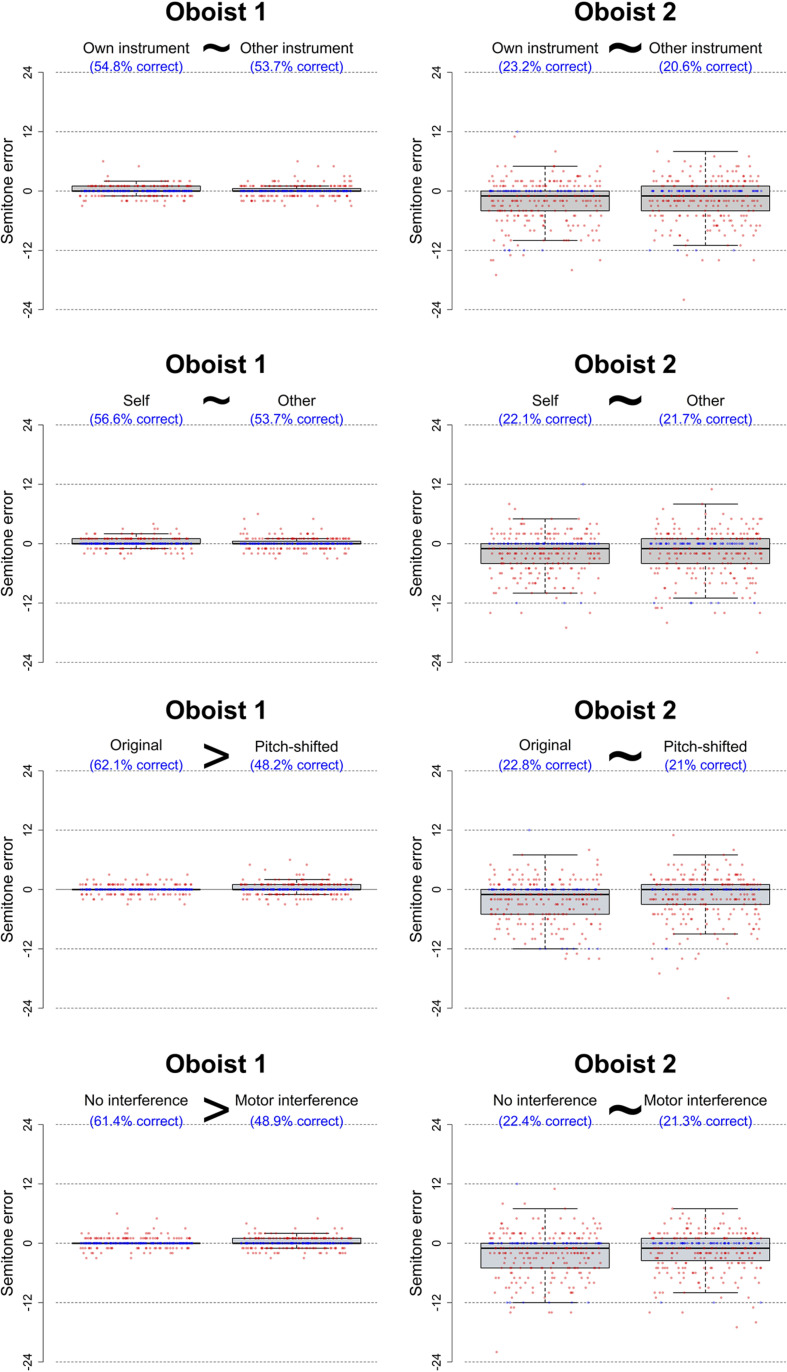
Boxplots with raw data of response deviation (in semitones) from the target pitch for correct (blue) and incorrect (red) pitch identification responses provided by the two oboists during Experiment B. The four sub-panels contain data from the four experimental manipulations: instrument (own instrument vs. other instrument), performer (self vs. other), transposition (original vs. pitch-shifted), and motor interference (no interference vs. motor interference). Percentage of correct responses in each condition is indicated in blue writing, and the box plots depict the number of semitones that responses were off from the target. The presence and direction of significant differences between conditions—as assessed with logistic regression modeling—are indicated with “>” signs whereas “∼” indicates that the relevant factor did not contribute significantly to the prediction of correct responses. As can be seen, whereas Oboist 2’s performance was not affected by any of the experimental manipulations, the use of pitch-shifted stimuli and the motor interference task decreased Oboist 1’s ability to correctly identify absolute pitch for oboe tones as well as increased the number of semitones that her responses were off from the target pitch.

Consistent with the results from Experiment A, one-sample proportions tests with continuity correction confirmed that both oboists identified pitch with an overall accuracy that was significantly above the theoretically motivated chance level (Oboist 1: 55.1% correct, 95% CI: 50.9–59.4%, χ^2^(1) = 1554.60, *p* < 0.0001; Oboist 2: 21.9% correct, 95% CI: 18.5–25.6%, χ^2^(1) = 128.82, *p* < 0.0001). Moreover, a Pearson’s chi-squared test with Yates’ continuity correction showed that accuracy was significantly higher for Oboist 1 than for Oboist 2 (χ^2^(1) = 125.76, *p* < 0.0001). Condition-wise percentage of correct responses is reported in Figure 5.

To investigate whether instrument, performer, transposition, and motor interference affected pitch identification performance to a significant extent, logistic regression analysis was conducted. Specifically, separate generalized linear models were fitted to the data from each of the two oboists using the *glm()* function from the “stats” package in R (R Core Team, 2018) with the four dichotomous condition variables as predictors. Interactions between these factors were not hypothesized and therefore not included in the initially specified models. Exploratory *post-hoc* analysis confirmed that adding interaction terms provided no significant improvement in fit to the data while merely increasing the BIC and AIC values.

As evident from the statistics reported in Figure 5 and Table 2, Oboist 1 made significantly more incorrect guesses when pitch-shifting was applied to stimuli and when she was asked to engage in the motor interference task. There was, on the other hand, no significant difference between the accuracy for stimuli recorded by herself vs. those recorded by the other oboist, or for stimuli recorded on her own oboe vs. those recorded on the other person’s instrument. The full model with all four predictors (AIC = 738.29) performed significantly better than a null model including only an intercept term (AIC = 750.37) (χ^2^(4) = 20.08, *p* = 0.0005).

**TABLE 2:** Main results from logistic regression of accuracy on the four condition variables: instrument (own oboe vs. other oboe), performer (self vs. other), transposition (original vs. pitch-shifted), and motor interference (no interference vs. motor interference), separately for Oboist 1 and Oboist 2.

	Oboist 1				Oboist 2			
	Coefficient [95% CI]	SE	*Z*	*p*	Coefficient [95% CI]	SE	*Z*	*p*
Intercept	0.72 [0.33, 1.11]	0.20	3.60	0.0003*	−1.10 [−1.56, -0.66]	0.23	–4.84	< 0.0001*
Instrument	−0.03 [−0.38, 0.31]	0.18	–0.18	0.8606	−0.15 [−0.56, 0.26]	0.21	–0.73	0.4679
Performer	0.12 [−0.22, 0.47]	0.18	0.70	0.4826	−0.02 [−0.43, 0.39]	0.21	–0.10	0.9173
Transposition	−0.58 [−0.93, −0.24]	0.18	–3.29	0.0010*	−0.11 [−0.52, 0.30]	0.21	–0.52	0.6039
Motor interference	−0.52 [−0.87, −0.18]	0.18	–2.95	0.0032*	−0.06 [−0.47, 0.34]	0.21	–0.31	0.7555

*^∗^indicates significance at α = 0.05.*

Excluding the conditions where Oboist 1’s performance was significantly impaired by the use of pitch-shifted tones or the motor interference task, she overall identified 90 out of 136 (66.2%) of the presented pitches accurately. Note that this is identical to her performance for oboe tones in Experiment A (also 66.2%). This suggests that she can on average identify two out of three pitches played on her own instrument type, and that this form of instrument-specific absolute pitch (ISAP) can be demonstrated with high levels of test-retest reliability.

Oboist 2’s performance was not significantly affected by any of the four experimental manipulations. A full model (AIC = 580.65) including instrument, performer, transposition, and motor interference as predictors predicted pitch identification accuracy no better than a null model with only an intercept term (AIC = 573.55) (χ^2^(4) = 0.90, *p* = 0.9239).

Oboist 2’s overall accuracy level (21.9%) was comparable to the one found in Experiment A across the two instrument types (24.3%). This suggests that her above-chance ability to identify pitches cannot be characterized as true ISAP and, therefore, was not negatively influenced by interference with any of the proposed strategies used by people with ISAP.

##### Semitone Error Values

Next, the extent to which absolute semitone error values were affected by the experimental manipulations was assessed. Given the highly skewed distribution of absolute semitone error values and because they are more properly regarded as ordinal than as continuous data, ordinal logistic regression analysis was conducted by fitting a proportional odds model with the *polr()* function from the “MASS” package in R (R Core Team, 2018). Consistent with the prior analysis, absolute semitone error values were modulo-12-transformed to assume octave equivalence.

The results from this analysis (Table 3) suggest that, for Oboist 1, transposition and motor interference interfered not only with accuracy in terms of the proportion of correct responses but also with the number of semitones that responses were away from the target. A likelihood ratio test demonstrated that the full model with all four predictors (AIC = 1121.24) significantly outperformed the null model with only intercept terms (AIC = 1134.08) (χ^2^(4) = 20.84, *p* = 0.0003). None of these effects were significant in the data from Oboist 2 where a full model (AIC = 2318.10) did not provide a better fit than the null model (AIC = 2311.29) (χ^2^(4) = 1.20, *p* = 0.8784).

**TABLE 3:** Main results from ordinal logistic regression of semitone off values on the four condition variables: performer (self vs. other), instrument (own oboe vs. other oboe), transposition (original vs. pitch-shifted), and motor interference (no interference vs. motor interference), separately for Oboist 1 and Oboist 2.

	Oboist 1				Oboist 2			
	Coefficient [95% CI]	SE	*t*	*p*	Coefficient [95% CI]	SE	*t*	*p*
Performer	−0.15 [−0.48, 0.18]	0.17	–0.88	0.3790	−0.06 [−0.36, 0.24]	0.15	–0.40	0.6895
Instrument	−0.03 [−0.36, 0.30]	0.17	–0.20	0.8421	0.08 [−0.22, 0.37]	0.15	0.52	0.6033
Transposition	0.59 [0.26, 0.93]	0.17	3.50	0.0005*	0.05 [−0.25, 0.34]	0.15	0.31	0.7571
Motor interference	0.47 [0.14, 0.80]	0.17	2.78	0.0055*	−0.12 [−0.42, 0.17]	0.15	–0.83	0.4082

**To save space, statistics for the intercept terms (6 for Oboist 1 and 11 for Oboist 2) are not shown here. Confidence intervals were obtained by profiling the likelihood function, and p-values were calculated by comparing the t-values against the standard normal distribution. ^∗^indicates significance at α = 0.05.**

##### Effects of Pitch-Shifting

To stimulate future *a priori* hypotheses to be tested in a larger sample of oboists or other instrumentalists, further exploratory analyses were conducted and will be reported in the next three subsections. First, we wanted to explore if there was a significant effect of the magnitude with which transposed pitches were shifted, even when we ignored the overall effect of notes being transposed. To this end, a logistic regression model with absolute pitch-shift interval as a continuous variable (in contrast to the binary variable used in the original analysis) was fitted specifically to Oboist 1’s data from trials with pitch-shifted stimuli. Indeed, there was a significant effect of pitch-shift interval (*coefficient* = −0.50, *SE* = 0.12, 95% *CIs*: [−0.73, −0.28], *Z* = −4.33, *p* < 0.0001). In light of this, we refitted the overall model for all Oboist 1’s trials with the continuous rather than binary pitch-shift variable. As evident from Table 4, absolute pitch-shift interval did indeed significantly predict correct responses. Furthermore, various information criteria and accuracy estimation with the data split into a training set of 67% and a test set of 33% of Oboist 1’s trials found that the revised model with absolute pitch-shift intervals (*accuracy* = 56.35%, *AIC* = 722, *BIC* = 744) slightly outperformed the original model with transposition as a binary predictor (*accuracy* = 53.59%, *AIC* = 738, *BIC* = 760).

**TABLE 4:** Main results from exploratory logistic regression of accuracy on the four condition variables with transposition operationalized as a continuous (rather than binary) variable describing the absolute number of semitones that pitch-shifted stimuli were transposed.

	Oboist 1			
	Coefficient [95% CI]	SE	*Z*	*p*
Intercept	0.82 [0.44, 1.21]	0.20	4.17	<0.0001*
Instrument	−0.03 [−0.38, 0.32]	0.18	–0.18	0.8584
Performer	0.13 [−0.22, 0.48]	0.18	0.71	0.4759
Transposition	−0.32 [−0.44, −0.20]	0.06	–5.11	<0.0001*
(absolute interval)				
Motor interference	−0.54 [−0.89, −0.19]	0.18	–3.00	0.0027*

**Only statistics for Oboist 1 who showed significant levels of instrument-specific absolute pitch (ISAP) are included.**

Thus, it appears that Oboist 1’s pitch identification performance deteriorated to an increasing extent the larger the intervals with which pitch was shifted. In interpreting these *post-hoc* findings, however, an important caveat calls for consideration. Specifically, because the pitch-shifting methodology outlined in Table 1 associates specific absolute pitches with specific interval transpositions, the current research design does not enable formal separation of pitch-specific and interval-specific effects. For example, the finding that pitch-shifting by 4 semitones leads to lower pitch identification performance than pitch-shifting by 1 semitone, could be (fully or partially) due to B♭3 (or any of the other pitches always shifted by 4 semitones) having more distinctive timbral features, thus leading to more frequent confusions when identifying pitch than for the specific pitches that were shifted by a single semitone.

##### Pitch-Specific Accuracy

**FIGURE 6:**
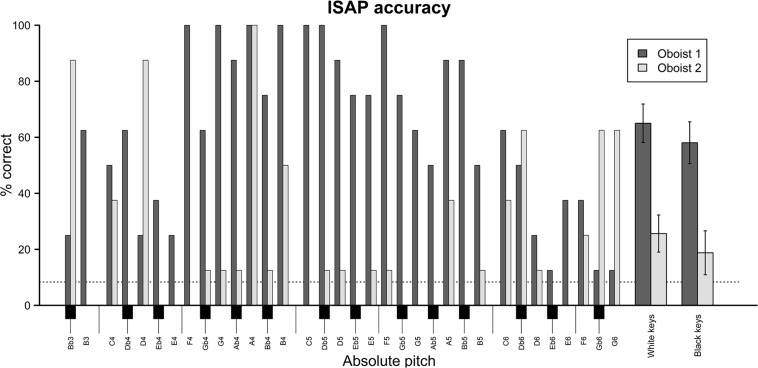
Bar plot indicating proportion of correct pitch identification responses (i.e., instrument-specific absolute pitch accuracy) for untransposed oboe tones separate for each of the two oboists and separate for each pitch level spanning the full range of the oboe from B♭3 to G6. Although only one enharmonic option occurs for each pitch level on the x-axis, response options included two enharmonic equivalents for each of the “black keys” (e.g., “C#4/D♭4” rather than merely “D♭4”). The bars on the far right depict proportion of correct responses for pitch categories corresponding to “white keys” (i.e., C, D, E, F, G, A, B) and “black keys” (C#/D♭, D#/E♭, F#/G♭, G#/A♭, A#/B♭) on the piano. Error bars represent standard errors of the mean.

Exploratory analysis on the accuracy of specific absolute pitch categories were also conducted. Specifically, descriptive statistics were computed for the two oboists in terms of the proportion of correct pitch identification responses separated by pitch level and by pitches corresponding to black and white keys on the piano keyboard (Figure 6). This endeavor was inspired, amongst others, by previous findings that global AP possessors identify white-key notes more accurately than black-key notes and show superior performance in the medium pitch register (Miyazaki, 1989). Because no interaction was assumed between motor interference and pitch level, responses with and without motor interference were included in this analysis. Since, however, transposition of tones following the system described in Table 1 cannot be assumed to have uniform effects across pitch levels, only responses to untransposed tones were featured.

As evident from Figure 6, mid-register pitches did indeed seem to be identified with somewhat greater accuracy than extreme-register pitches by Oboist 1 whereas Oboist 2 showed no such tendency. Oboist 2’s performance was around chance level for most pitch levels with an accuracy of 50% or above for specific (mostly extreme-register) pitches like B♭3, D4, A4, B4, D♭6, G♭6, and G6. Interestingly, there was no clear overlap between the pitches identified exceptionally well by each of the two oboists. A remarkable exception was the orchestral tuning note, A4, which was the only pitch identified with 100% accuracy by both participants. There was, moreover, an overall numeric (albeit not statistically significant) tendency toward more accurate performance for white-key notes than for black-key notes. This tentatively suggests that ISAP accuracy may be enhanced by exposure, but we note that this tentative effect did not seem more strongly present in Oboist 1 than in Oboist 2 as would have been expected had this indeed been the case. Thus, while nothing definitive can be concluded based on these preliminary observations, they do call for future investigation.

##### Post-experiment Questions

It should be noted that Oboist 1 held a belief prior to the experiment that she had ISAP. After participating in the experiment, the concept of ISAP was described to Oboist 2, and she was asked whether she had ever thought she experienced ISAP, to which she responded no. Oboist 2 was also asked if she thought she had performed better identifying oboe tones over piano tones, to which she also responded no.

Participants were asked to type answers to open-ended questions about strategies, fatigue, perceived difficulty, and confidence. Both oboists related their strategies for identifying notes played on the oboe to fingerings. Oboist 1 described using kinesthetic imagery of the note that she thought she heard, replaying the note while imagining the fingering and deciding whether this felt matched or mismatched. In more uncertain cases, she would try two or three different fingerings to see which felt like the best fit to the sound. She noted that this strategy was more difficult to use in the motor condition. Oboist 2 described listening for perceived resistance (back pressure) in relation to the number of fingers that would be put down with each note. While Oboist 1 expressed some frustration with the motor condition, Oboist 2 reported that she felt more confident in the motor condition (though note this did not result in significantly increased performance). Both oboists also described using timbral cues to identify oboe tones, with Oboist 1 referencing “roundness,” “brightness,” “darkness,” and “texture” and Oboist 2 referencing “quality,” “resonance,” and “color.” Strategies for identifying piano tones differed. Oboist 1 described using registral timbre and visualization of the keyboard; Oboist 2 imagined the piano notes played on the oboe.

## Discussion

Here, we presented a novel theory of instrument-specific absolute pitch (ISAP), predicting a pertinent role for instrument-specific timbral cues and motor imagery in enabling above-chance absolute pitch identification in some expert musicians. Preliminary data from two proof-of-concept case studies were collected and analyzed. While not providing any complete, formal test of our theory, these were intended to serve as methodological guidelines for how this can be achieved in the future. In Experiment A, the two oboists were able to identify both oboe and piano tones significantly above chance. Because Oboist 1 identified pitch for oboe tones significantly better than for piano tones, she appears to have ISAP whereas Oboist 2 does not. Thus, as is the case for global absolute pitch (AP), it seems that some musicians may have ISAP while others do not. In Experiment B, Oboist 1 performed significantly worse when identifying tones that had been pitch-shifted and when engaging in a motor interference task. Oboist 2 did not demonstrate such differences, and neither oboist demonstrated significantly different performance as a function of performer or instrument. For both experiments, Oboist 1’s responses were not only more accurate, but her errors also exhibited smaller variance around the target pitch.

The idea that certain timbres are associated with increased accuracy in pitch identification has parallels in global AP. Baharloo et al. (1998) distinguished various types of AP, identifying a group of people (AP-4) who can identify pitches played on piano but not sine tones. They suggested that the mechanisms for this group may differ from other AP possessors in that they benefit from timbre or harmonics in pitch identification. Because primary instrument was not assessed in this study, it is unclear whether AP-4 individuals demonstrated ISAP for piano or simply relied on harmonic complexity. While above-chance performance for piano may result from experience with the piano timbre, it may also be related to generalized pitch category knowledge, which Heald et al. (2014) found is modulated by musical experience. Our study contributes to this topic by providing preliminary empirical substantiation that timbre sometimes plays a more prominent role in AP, which may manifest as ISAP.

We have operationalized ISAP as significantly more accurate pitch-naming performance for the timbre of one’s primary instrument than for other instruments, regardless of the size of this effect. Future studies with larger sample sizes and more diverse recruitment should investigate the prominence and range of effect sizes for ISAP and assess systematic differences between different musical instruments. Such data will answer whether different subtypes of ISAP may exist, as has been argued for global AP (Baharloo et al., 1998, 2000). Additionally, full-scale studies with participants naive to the experimental hypotheses will resolve concerns about demand characteristics resulting from the inclusion of Oboist 1 as a case-study participant.

If some musicians have ISAP while others do not, we might examine the etiologies suggested for global AP as candidate etiologies for ISAP. A learning or experience component seems germane to ISAP given the posited pitch-naming advantage for a musician’s primary instrument(s) of expertise. At the time of the experiments reported here, the two oboists had played oboe for 19 and 13 years, respectively; it remains possible that the 6-year difference in experience may contribute to Oboist 1’s advantage, though it is unknown when exactly her ISAP developed. Oboist 1 began playing oboe at age 10, while Oboist 2 began at age 12. This small-scale study does not offer sufficient information to investigate whether a critical period exists for ISAP as has been argued for global AP (Deutsch et al., 2006; Gervain et al., 2013; though see Wong et al., 2019, and Van Hedger et al., 2019, for evidence of AP acquisition in adulthood).

Another potential predictor of ISAP is training in solfège, which has similarly been implicated in global AP (Wilson et al., 2012). Indeed, Oboist 1 specialized in fixed-do solfège and has continued using it whereas Oboist 2 specialized in the moveable-do system and ceased practicing it after formal instruction ended. Future research should investigate whether ISAP is more common in individuals trained in fixed-do compared to movable-do solmization.

In global AP, Miyazaki (1989) found that AP possessors more accurately identified pitches associated with white than black keys on the piano keyboard as well as identified pitches in the middle range better than pitches in the extreme ranges. This suggests a potential role for implicit learning in the acquisition of absolute pitch. Our study provides some evidence consistent with the assertion that such learning effects may extend to ISAP. Indeed, Oboist 1 performed relatively better with mid-range pitches whereas Oboist 2, who does not have ISAP, did not display such mid-range advantage. Both oboists trended non-significantly toward better performance on white-key over black-key pitches. For untransposed tones, both oboists labeled A4, the orchestral tuning pitch, with 100% accuracy (8 out of 8 correct). Strong associations between this pitch and its label are regularly reinforced through rehearsals and performances where it is most often the principal oboist’s job to provide the tuning pitch for the rest of the ensemble.

Another possible contribution to ISAP inspired by AP etiology is genetics (e.g., Baharloo et al., 1998, 2000; Gregersen et al., 1999; Theusch et al., 2009). For global AP, it has been argued that genetics may influence pitch identification performance via cognitive style (Chin, 2003), though Schlemmer (2009) did not find support for all aspects of this hypothesis. Still, we might consider whether differences in cognitive style influence which properties of timbre individuals primarily attend to during practice and listening, with possible implications for the development of ISAP ability.

In Experiment B, Oboist 1 performed significantly better with untransposed tones than with artificially pitch-shifted tones, suggesting that pitch-specific timbral idiosyncrasies may assist in ISAP. *Post-hoc*, we assessed whether Oboist 1 tended to mislabel transposed tones as their original, untransposed pitch, but we did not establish any systematic pattern in responses. Although efforts were made to avoid artifacts, it is possible that the pitch-shifting method created subtle alterations in timbre which could have masked the telltale idiosyncrasies of specific pitch categories. Yet, it remains surprising that Oboist 1 performed better for pitch-shifted oboe tones than for untransposed piano tones. This suggests that the mere sound of the oboe activates absolute pitch templates that only partly rely on pitch-specific idiosyncrasies. Because transposed and untransposed pitches were mixed within each block, it is also possible that Oboist 1’s relative pitch influenced identification of transposed tones. That is, when an unfamiliar (transposed) note was played following a particularly obvious, untransposed note, an automatic reliance on relative pitch may have come into play. The use of such response strategies should be systematically assessed in future experiments through open-ended post-experiment questions.

Oboist 1, moreover, performed significantly worse when engaging in motor tasks (chewing gum and wiggling fingers), thus supporting the hypothesis that articulatory motor imagery is involved in ISAP. Follow-up experiments will need to compare oboe-specific motor interference with generalized motor distraction using body parts not directly involved in oboe playing to further substantiate this finding.

Another open question regarding motor imagery is its availability to musicians as a pitch identification tool. Given instructions to attend to kinesthetic feelings evoked by tones, might Oboist 2 be able to learn to use this mechanism? Future research might productively borrow methodology from Wong and Wong (2014), who had individuals hold their instrument and finger the pitch that they thought they were hearing. We might hypothesize that individuals with ISAP and possibly also those not demonstrating significant ISAP in an initial test would identify pitch more accurately in this instrument-fingering condition.

Further understanding of the motor imagery mechanism may be obtained using neuroimaging and brain stimulation techniques. We would hypothesize that when identifying pitches on their primary instrument, individuals with ISAP would show greater activity in lip- and hand-related sensorimotor areas than individuals without ISAP and that brain stimulation may affect these processes. Choi et al. (2015) observed structural and functional differences in wind players, and structural and functional differences have also been observed between those with and without global AP (e.g., Zatorre et al., 1998; Keenan et al., 2001; Leipold et al., 2019; Matsuda et al., 2019). It seems reasonable to predict parallel neural correlates of ISAP.

Regarding response strategies in naming pitches more generally, Letailleur et al. (2020) found a rich diversity of approaches to identifying the pitch of piano tones that combined auditory, visual, and kinesthetic strategies. Some of the strategies that they identified seem to be reflected in answers to the post-experiment questions collected in the current study, specifically “view of a musical instrument or view of the body position required to play that note,” “note recognition by association of pitch with specific auditory hues, or the notion of ‘timbre-pitch,’ ” and “feeling of an instrumental gesture associated to the production of a note.” The findings of Letailleur et al. (2020) demonstrate that musicians use a wide variety of strategies in note identification tasks and suggest that future research on the mechanisms of instrument-specific absolute pitch may benefit from analysis of participants’ accounts of their own strategies.

Having observed only a single oboist with ISAP, the lack of supporting evidence for the performer and instrument hypotheses does not definitively disprove them. Therefore, these secondary hypotheses do remain open for future research. Nonetheless, it is pertinent to note that, despite general timbral differences among different players and instrument exemplars (Chudy, 2016), we have not encountered anecdotal evidence consistent with the performer hypothesis and only a single case of superior pitch identification for one’s own instrument exemplar (Whipple, 1903); rather, these hypotheses were formed as implications of the instrument-type hypothesis. While we cannot dismiss effects of performer or instrument for some musicians, it is likely that performer or instrument idiosyncrasies are not perceptible or useful enough to aid in pitch identification at the ISAP population level.

Moving forward, therefore, we reframe our theory of ISAP to include two proposed mechanisms: articulatory motor programming and the use of pitch-specific timbral idiosyncrasies for a given instrument type. For experienced musicians with considerable motor experience on a particular instrument, these theorized mechanisms may be related. On one hand, the general timbre of a musician’s instrument may be enough to activate motor planning that can assist in pitch identification. On the other hand, pitch-specific timbral idiosyncrasies may either be necessary for or enhance pitch identification. Timbral idiosyncrasies may also be used to identify pitch without motor experience: in particular, this would suggest that non-musicians can learn ISAP for a given non-primary instrument. Such hypotheses may be empirically tested. Our theory, moreover, predicts that ISAP possessors may not always rely on the same mechanisms or strategies as global AP possessors. Future research will need to assess variance in the strategies that other ISAP possessors use.

In sum, this proof-of-concept study indicates that ISAP is detectable in some musicians, but not in others, and may involve pitch-specific timbral cues and motor imagery. This implies that future experiments must first identify individuals with ISAP before testing for underlying mechanisms and generalizing findings to wider populations of oboists and other instrumentalists. No evidence was found in support of the performer-specific or instrument token-specific sub-hypotheses. This suggests that ISAP typically generalizes beyond a specific token instrument or player. Consequently, we suggest that future research can likely use generalized stimuli not recorded by study participants without risking compromising the effects. By testing only factors significant for Oboist 1 (instrument-type and articulatory motor planning), the total experiment duration can be reduced dramatically to facilitate practical feasibility with a larger sample. A deeper understanding of ISAP would have implications for teaching, practicing, musicianship tasks, and the understanding of musical expertise. Musicianship tasks like dictation are almost exclusively given on the piano; the existence of ISAP would suggest that timbres should be diversified in the aural skills classroom and possibly even individually tailored according to main instrument. If ISAP can be developed with practice, it would be advantageous to educate students about the skill, which might become useful in tasks such as transcription, and to incorporate embodied procedures to solving dictation and transcription exercises. Such practice would ultimately capitalize more effectively on the embodied aspects of musicianship and musical skill acquisition (Schiavio and van der Schyff, 2018).

## Data Availability Statement

The raw data supporting the conclusions of this article will be made available by the authors, without undue reservation, to any qualified researcher.

## Ethics Statement

The studies involving human participants were reviewed and approved by the Ohio State University Institutional Review Board. The participants provided their written informed consent to participate in this study.

## Author Contributions

LR and NH developed the theory, designed the case studies, conducted statistical analysis, and co-wrote the manuscript. NH edited the stimuli, configured the stimulus presentation software, and produced the figures and tables. LR collected the data. Both authors contributed to the article and approved the submitted version.

## Conflict of Interest

The authors declare that the research was conducted in the absence of any commercial or financial relationships that could be construed as a potential conflict of interest. The reviewer SM declared a shared affiliation with one of the authors, LR, to the handling editor at the time of review.

## Footnotes

1 We use the term “tendency” here to acknowledge that while a particular instrument’s timbre or intonation may tend to vary in certain contexts, the player is often able to counteract these tendencies to at least some extent. Therefore, while tendencies in timbre and intonation may be present on the instrument as a whole, they are not always audible.
